# Assessing accuracy and specificity of faecal source library for microbial source-tracking, using SourceTracker as case study

**DOI:** 10.1093/bioadv/vbaf103

**Published:** 2025-04-29

**Authors:** Timothy J Y Lim, Yussi M Palacios Delgado, Anna Lintern, David T McCarthy, Rebekah Henry

**Affiliations:** Department of Civil & Environmental Engineering, Monash University, Clayton, VIC 3800, Australia; Department of Civil & Environmental Engineering, Monash University, Clayton, VIC 3800, Australia; Department of Civil & Environmental Engineering, Monash University, Clayton, VIC 3800, Australia; School of Environmental Sciences, University of Guelph, Guelph, ON N1G 2W1, Canada; Department of Civil & Environmental Engineering, Monash University, Clayton, VIC 3800, Australia; School of Public Health & Preventive Medicine, Monash University, Melbourne, VIC 3004, Australia

## Abstract

**Motivation:**

Understanding the quality of the source library prior to undertaking library-dependent microbial source-tracking (MST) is an essential, but often overlooked, primary analysis step.

**Results:**

We propose an assessment approach to validate the quality of amplicon-derived faecal source libraries. This approach was demonstrated on a faecal source library consisting of 16S rRNA paired-end amplicon sequences, obtained from various animal types in Victoria, Australia. First, a leave-one-out (LOO) analysis was performed to assess the accuracy of source category groupings by identifying the number of samples incorrectly assigned to a different source category (i.e. animal type). Following a quality control procedure to decide retaining/removing/grouping incorrectly assigned samples, we then assessed if the sample sizes for each source type were sufficient to properly characterize the source fingerprints. Results from LOO demonstrated 15.5% of samples were incorrectly assigned, with high error rates in birds and wallabies within our source library. Increasing the sample size improved source identification accuracy. However, accuracy eventually plateaued in a source-specific manner. Importantly, this highlights the importance of conducting thorough assessments to understand the quality and limitations of the source library prior to library-dependent MST applications.

**Availability and implementation:**

QIIME2 is available via https://qiime2.org/; SourceTracker v2.0.1 is available via https://github.com/caporaso-lab/sourcetracker2; Pipeline for LOO is available via https://github.com/MonashOWL/Bioinformatics-IlluminaMGI/tree/main/16S/LOO; Pipeline for sample size assessment is available via https://github.com/MonashOWL/Bioinformatics-IlluminaMGI/tree/main/16S/Source%20variability.

## 1 Introduction

Source apportionment techniques such as microbial source-tracking (MST) are growing in popularity as they have wide applications. One such applications is for the monitoring of water quality, where that they can be used to infer the source of contamination within complex catchments (Raza *et al.* 2021). Microbial community fingerprinting, also known as community-based MST via 16S rRNA amplicon sequencing is a library-dependent method whereby a library of microbial communities is derived for faecal sources (e.g. faecal matter of different animals) and sink environmental samples (e.g. downstream water samples). Assuming that these sources and sink samples contain their own distinct microbial community composition (or microbial community fingerprint), a mixing model [e.g. SourceTracker (Knights *et al.* 2011)] is used to define the contribution of each faecal source to the sink sample under investigation (Raza *et al.* 2021).

Microbial community fingerprinting and library-dependent MST are increasingly used, with the potential to inform regulatory stakeholders. Establishing standardized methods and best practices is particularly important as researchers increasingly rely on publicly available datasets, such as those in the Sequence Read Archive (SRA), to expand their source libraries.

There are recognized considerations to note when using public domain data. First, users should be aware to use only datasets that are regionally-specific and native to their area of study, or at least ensure that there are native sources within the source library which may be composed of non-native sources (Unno *et al.* 2018, Brown *et al.* 2019, Barrett *et al.* 2025). This is due to the geographical variability-induced uncertainties in faecal communities of different regions, which can impact the accuracy of the results when the library is only composed of non-native sources (Staley *et al.* 2018). A second consideration is temporal factors (e.g. where a single animal species or environment is sampled over time). This may result in shifts in microbiome, which alter assay specificity and impacting the representativeness of faecal microbial community from a particular animal species (O’Dea *et al.* 2019). However, the temporal stability of faeces differs between different animals, with some animals (e.g. dogs) having relatively greater stability over the others (e.g. migratory birds) (O’Dea *et al.* 2019, Thie *et al.* 2022). As a result, this can be concerning, especially in the case where the design of a study requires collection of animal faeces across a few years. In addition, it was also important to consider the impacts from the number of samples available for each source type in the library, including how they can affect representativeness of a source type, which can impact identification accuracy (IA) of community-based MST.

Due to the above considerations and associated introduction of analytical uncertainty, it is paramount to first assess the source library before commencing library-based MST. Formulating best practices would improve confidence in result calculation and align the amplicon-based approach with other library-based methods, which have employed jack-knife analysis for accuracy (Stoeckel and Harwood 2007), or test for representativeness of phenotypic or genetic diversity in indicator organism populations (Mott and Smith 2011). Thus, the aim of this study is to provide a pipeline for assessing the quality (i.e. accuracy) of the source library to be used in library-dependent MST and understand if the source library has enough samples to represent sources. In this study, we focus on SourceTracker v2 (Knights *et al.* 2011) as the algorithm used to conduct community-based MST in assessing quality of source library.

This pipeline uses the leave-one-out (LOO) method for cross-validation, followed by assessment of whether the faecal source library contains enough samples to accurately represent the faecal sources. In relation to LOO, the strategy has been previously tested to identify predictive performance of SourceTracker in differentiating between ecotypes using sequence abundance of antibiotic resistance genes (Li *et al.* 2018). However, within the nature of complex natural environments, where it is expected to have heterogeneous datasets of animal faeces, the application of LOO offers distinct advantages in quality assessment of samples prior to analysis. The LOO method identifies source categories by analysing the core microbiome characteristics of faeces. Its iterative approach across all samples in the source library minimizes spatial and temporal biases while ensuring assay specificity.

## 2 Methods

A demonstration dataset (SRA project reference PRJNA309092) was applied to develop and refine the proposed pipeline (Henry *et al.* 2016). This dataset was selected due to the diversity of faecal sources from bat, bird, cat, chicken, cow, deer, dog, horse, human (sewage), kangaroo, possum, rabbit, rat, sheep, wallaby, waterbird, and wombat (Table 1). To account for geographic stability and regional specificity of the sources, faecal samples were freshly collected in the region of Victoria, Australia. Pipelines for processing of 16S rRNA amplicon data derived from the faecal samples using QIIME 2 version 2023.9 (Bolyen *et al.* 2019), and the pipeline for assessing the quality of the source library were documented in Lim (2024).

**Table 1.** Analysis results from LOO analysis and uncertainties from number of source samples included.

Animal	Total samples	Samples incorrectly assigned in LOO	Sample size needed for ≥90% IA
Bat	17	0	4
Bird	9	5	Maximum 45% IA
Cat	18	1	10
Chicken	16	0	2
Cow	40	6	12
Deer	26	5	24
Dog	20	4	17
Horse	14	0	3
Human	29	1	2
Kangaroo	9	2	8
Possum	17	4	16
Rabbit	17	2	7
Rat	19	1	2
Sheep	4	1	Maximum 80% IA
Wallaby	20	13	Maximum 50% IA
Waterbird	44	7	30
Wombat	30	2	23
**Total**	349	54	–

To assess the quality of the faecal source library, we first quantified the error rate of source identification using LOO analysis of faecal samples. For this, each faecal sample was assigned as a sink, one at a time, with the remaining faecal sample(s), irrespective of the type of faecal matter, being assigned as sources. SourceTracker (v2.0.1) was then conducted using default parameters, with a rarefaction depth of 2000. Additionally, as per the general procedure described in Henry *et al.* (2016), five replicate runs were performed, with the results averaged, and relative standard deviation (RSD) calculated [n.b., the SourceTracker version used in Henry *et al.* (2016) was v0.9.8. Despite the difference in SourceTracker versions, SourceTracker v2.0.1 replicates the functionality of the original SourceTracker package by Knights *et al.* (2011), with extended parallel processing functionality]. The results were then sorted and categorized according to the type of animal, with results containing RSD ≥100% ignored. Based on the results, a SourceTracker run was defined as erroneous when the animal type of the highest source contributor was different from the animal type of the sink sample. The error rate of source identification was then calculated by calculating the ratio between the total number of erroneous cases against the total number of SourceTracker runs in the analysis.

Subsequently, the following quality control (QC) consideration points were taken into account for erroneous samples to decide if the samples should be retained, removed, or grouped with other animals: (i) if known—sample was collected by a trained and experienced personnel in identification of different animal faecal matter; (ii) identify possible reasons for observed microbiome shift based on metadata availability (e.g. moisture content, age of sample, or presence of diseased state in animal); (iii) if most of the samples from an animal were commonly misassigned to another animal type (and vice versa), conduct beta-diversity analyses to identify if the two animal groups are significantly different from each other; and (iv) determine if any of these commonly misassigned animal groups poses a significant faecal input risk. From these, it can then be decided whether to retain/remove incorrectly assigned samples, or group them with the animal group that was commonly misassigned to for further analyses, depending on the context.

The following process was conducted to determine whether the faecal source library contained enough samples to accurately represent the faecal sources and to identify the sample size at which source characterisation should be undertaken. For this, samples were randomly drawn from the total number of samples (*N*) for each animal, beginning at *n* = 1 to *n* = *N* −1, and allocated to be source samples in the modelling run. All remaining samples (i.e. *N* −*n*) were designated as sink samples within the associated metadata file. The random sampling process was repeated 10 times (with replacement) for each number of sources (i.e. 10 number of draws with replacement for each number of sources). Samples from all the other animals were included as sources for all model runs (e.g. for a modelling run that is intended to identify the number of rabbit samples required, the other animals would be also included in that SourceTracker modelling run). An illustrative example of this process is provided (Supplementary Fig. S1).

SourceTracker (v2.0.1) analysis was then conducted as described above (i.e. five replicate runs with results averaged and RSD calculated). Similarly, to LOO analysis, the results were categorized according to the type of animal, with erroneous cases identified. In relation to that, for each sample size, we calculated the IA in source apportionment for each animal and the average IA across all animal types. This was based on the ratio of number of cases where the correct animal was successfully identified as the source to the total number of cases.

## 3 Results

LOO analysis using demonstration dataset revealed that 15.5% of the samples were misclassified to other source animals, based on the animal with the highest source contribution (53 out of 354 samples; Table 1). Specifically, there was a very high error rate for birds (5 out of 9 incorrectly assigned) and wallabies (13 out of 20 incorrectly assigned). We noted that majority of the incorrectly assigned birds and wallabies were assigned as waterbirds (40%) and wombats (∼62%), respectively.

We then examined the incorrectly assigned samples according to the defined QC consideration points. Using birds and wallabies as examples, as these samples contained relatively higher error rates in LOO, it was observed that if collection by trained personnel is assumed positive, it was the secondary definition of the source type that may have introduced the greatest uncertainty. For example, during collection of bird samples, the definition of bird was not limited to certain species only. Therefore, it is expected that the faecal signatures would have large known variations in gut microbiome associated with food intake (Davidson *et al.* 2020). This was further supported by the results from beta-diversity analyses, including Jaccard, Bray-Curtis, and unweighted UniFrac distances showed that there were significant differences between birds and waterbirds (*P* < .05). Similar results from beta-diversity analyses were observed for wombat and wallaby cross-assigned samples. Based on these findings, the next step is to determine whether they represent a significant faecal input risk within the catchment, before deciding whether to retain or remove incorrectly assigned birds and wallabies, or to group them with waterbirds and wombats, respectively, for downstream analyses.

**Figure 1.**
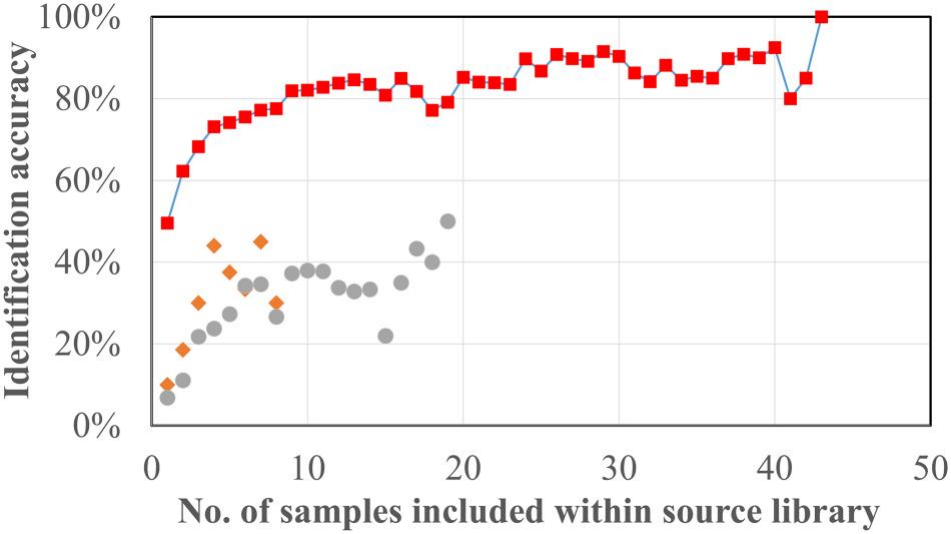
Plot of IA against number of samples included within source library for each animal. For the purpose of discussion, we only included IA for birds (represented using diamond/orange marker), wallabies (represented using circle/grey marker), and average of all source types (represented using square/red marker with line) in this plot. For more information regarding IA of other animals, refer to Supplementary Fig. S2.

When evaluating whether the source library contained an adequate number of samples to represent sources using the demonstration dataset, it was observed that increasing the number of samples for a particular source (e.g. animal) improved the accuracy of SourceTracker-predicted contributions (Fig. 1; refer to Supplementary Fig. S2 for more details). However, the IA for all the sample types eventually reached a source-specific plateau, in which inclusion of more samples did not significantly improve the IA. Based on this, the number of source samples required to achieve ≥90% IA was determined. While some sources reached ≥90% IA with as few as *n* = 2 samples, certain animals did not achieve ≥90% IA even with the maximum sample size (Table 1). Specifically, the IA for birds (highest 45% at *n* = 7) and wallabies (highest at 50% at *n* = 19) were much lower than expected. While collecting more samples for these animals may increase the IA, it should be noted that there will be higher uncertainties in source-tracking results for birds and wallabies as identified previously.

## 4 Discussion

Users should provide considerations for geographical and temporal variability during preparation of source library for library-dependent MST (Staley *et al.* 2018, O’Dea *et al.* 2019). However, it is also essential to assess the source library in terms of both accuracy and specificity, though not often reported within literature (Brown *et al.* 2019). As SourceTracker works by employing Bayesian prior probabilities to infer the likelihood that a taxon is being originated from a source (Knights *et al.* 2011), this may result in errors in source identification if different animal types have similar gut microbial community compositions, or live within constrained ecosystems (e.g. urban informal settlements).

The results demonstrated that the decision to retain, exclude, out-group, or group samples from two or more animal types for further analysis should depend on the outcome of the outlined QC consideration points and the downstream application. For example, in the case of birds, the incorrect assignments were likely to be the result of species out-groupings. Furthermore, there were significant differences between birds and waterbirds from beta-diversity analyses. Based on these information, it is important to consider the application context before deciding the action in dealing with the incorrectly assigned samples. For instance, when applying the source library as part of human-derived faecal contamination studies, it may be worth grouping these samples generally as birds.

Nevertheless, whilst the observations from the pipeline were true for the demonstration library we used for this study, the observations were likely to be different when other source libraries were used. Importantly, these systematic assessment pipeline in combination highlighted the importance of conducting thorough source library evaluation, which can be applied across any library-dependent MST methods. This enables users to understand the quality and limitations of their source library, including the related uncertainties on the source sample size, prior to further downstream library-dependent MST analysis. Furthermore, this study provided insights in resource allocation related to the collection and sequencing of faecal samples for generation of regional source libraries. Without evaluation of source library, users will not be able to understand the errors and uncertainties exhibited from each of the sources, leading to possible detrimental impacts related to the interpretation of library-dependent MST results.

## Supplementary Material

vbaf103_Supplementary_Data

## Data Availability

The data underlying this article are available in Sequence Read Archive at https://www.ncbi.nlm.nih.gov/sra/, with project reference PRJNA309092. Test datasets (subset of original dataset) are also generated as examples to help users better understand the proposed quality assessment procedures, which are available at https://github.com/MonashOWL/Bioinformatics-IlluminaMGI/tree/main/16S/LOO/Demo (for LOO) and https://github.com/MonashOWL/Bioinformatics-IlluminaMGI/tree/main/16S/Source%20variability/Demo (for sample size assessment).
