# Supplementary material for: Assessing accuracy and specificity of faecal source library for microbial source-tracking, using SourceTracker as case study
Source: Bioinform Adv. 2025 Apr 29;5(1):vbaf103. doi: 10.1093/bioadv/vbaf103 (PMC12092083; doi:10.1093/bioadv/vbaf103)
Supplement: vbaf103_Supplementary_Data [file vbaf103_supplementary_data.zip › SourceTracker_source_library_QC_Supplementary materials.docx]

**Assessing accuracy and specificity of faecal source library for microbial source-tracking, using SourceTracker as case study - Supplementary materials**


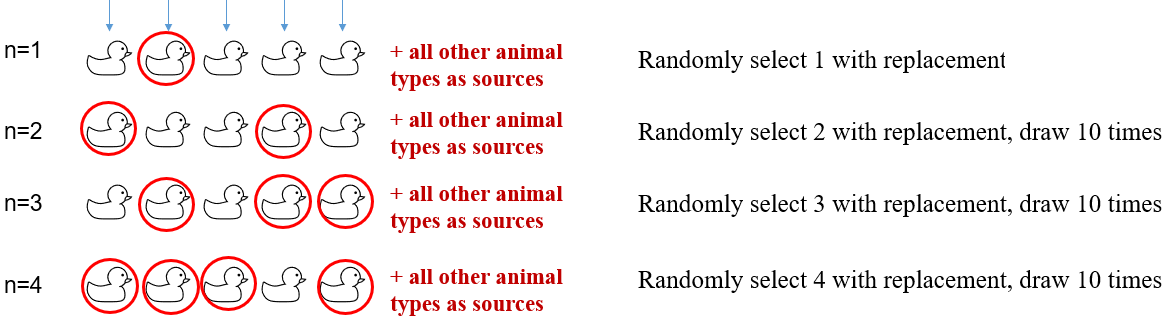


**Figure** **S1.** Illustration of random sampling process, using duck with a total of five number of samples (sum of source and sink samples). There are five a total of five duck samples (i.e., N = 5). Among these five ducks, at number of sources = 1 (denoted using n), only one duck was randomly selected to be the source samples (i.e., could be any one of the blue arrows), while the rest of the ducks were be assigned as sink samples. SourceTracker was then used to determine whether the ducks in sinks can be correctly matched to the sources by only using one duck as the source, together with sources from other animal types. This process was repeated 10 times. Due to the replacement rule, some of the ducks may be selected more than once. Subsequently, at number of sources = 2 (i.e., n = 2), two ducks were randomly selected as sources with replacement, with the remaining as sink samples. This was followed by SourceTracker modelling runs, and the process was repeated 10 times. The process was similar for n = 3 and n = 4 until n equals to total number of ducks minus 1 (i.e., N-1). This was due to all five ducks cannot be assigned as the sources, or else there would be no sinks

**Figure** **S2.** Plot of identification accuracy against number of samples included within source library for each animal type. The identification accuracy is determined based on the ratio of the number of times the biggest source contributor was identified to be the same as the sink, to the total number of cases for each number of sources. The blue line with red square marker represents the average (mean) identification accuracy in source apportionment (i.e., mean of identification accuracy in source apportionment across all animals)
